# Infection of *Anopheles aquasalis* from symptomatic and asymptomatic *Plasmodium vivax* infections in Manaus, western Brazilian Amazon

**DOI:** 10.1186/s13071-018-2749-0

**Published:** 2018-05-04

**Authors:** Keillen M. Martins-Campos, Andrea Kuehn, Anne Almeida, Ana Paula M. Duarte, Vanderson S. Sampaio, Íria C. Rodriguez, Sara G. M. da Silva, Claudia María Ríos-Velásquez, José Bento Pereira Lima, Paulo Filemon Paolucci Pimenta, Quique Bassat, Ivo Müller, Marcus Lacerda, Wuelton M. Monteiro, Maria das Graças V. Barbosa Guerra

**Affiliations:** 1 Programa de Pós Graduação em Medicina Tropical, Escola Superior de Ciências da Saúde, Universidade do Estado do Amazonas, Manaus, Brazil; 2 Departamento de Ensino e Pesquisa, Fundação de Medicina Tropical Dr. Heitor Vieira Dourado, Manaus, Brazil; 3 ISGlobal, Barcelona Centre for International Health Research (CRESIB), Hospital Clínic-Universitat de Barcelona, Barcelona, Spain; 4 Fundação de Vigilância em Saúde, Manaus, Amazonas Brazil; 5 Instituto de Pesquisas Leônidas & Maria Deane, Fiocruz, Manaus, Brazil; 6 Instituto Oswaldo Cruz, Fiocruz, Rio de Janeiro, Brazil; 7 Centro de Pesquisas René Rachou, Fiocruz, Belo Horizonte, Brazil; 8 Walter and Eliza Hall Institute, Parkville, Australia

**Keywords:** Malaria, Gametocytes, Vector, Elimination, Membrane feeding assay

## Abstract

**Background:**

Asymptomatic individuals are one of the major challenges for malaria elimination programs in endemic areas. In the absence of clinical symptoms and with a lower parasite density they constitute silent reservoirs considered important for maintaining transmission of human malaria. Studies from Brazil have shown that infected individuals may carry these parasites for long periods.

**Results:**

Patients were selected from three periurban endemic areas of the city of Manaus, in the western Brazilian Amazon. Symptomatic and asymptomatic patients with positive thick blood smear and quantitative real-time PCR (qPCR) positive for *Plasmodium vivax* were invited to participate in the study. A standardised *pvs25* gene amplification by qPCR was used for *P. vivax* gametocytes detection. *Anopheles aquasalis* were fed using membrane feeding assays (MFA) containing blood from malaria patients. Parasitemia of 42 symptomatic and 25 asymptomatic individuals was determined by microscopic examination of blood smears and qPCR. Parasitemia density and gametocyte density were assessed as determinants of infection rates and oocysts densities. A strong correlation between gametocyte densities (microscopy and molecular techniques) and mosquito infectivity (*P* < 0.001) and oocysts median numbers (*P* < 0.05) was found in both groups. The ability to infect mosquitoes was higher in the symptomatic group (41%), but infectivity in the asymptomatic group was also seen (1.42%).

**Conclusions:**

Although their infectivity to mosquitoes is relatively low, given the high prevalence of *P. vivax* asymptomatic carriers they are likely to play and important role in malaria transmission in the city of Manaus. The role of asymptomatic infections therefore needs to be considered in future malaria elimination programs in Brazil.

**Electronic supplementary material:**

The online version of this article (10.1186/s13071-018-2749-0) contains supplementary material, which is available to authorized users.

## Background

Despite large reductions in burden in the least two decades [1], malaria remains one of the most important public health problems worldwide. Overall, it is estimated that 3.2 billion people in 97 countries and territories are at risk of being infected with *Plasmodium* species and developing the disease [1]. According to the World Health Organization (WHO), 214 million cases of malaria and 438,000 deaths were recorded in 2015 [1]. In Brazil, from January to November 2016, 114,287 malaria cases were recorded [2]. In the same period, the Amazonas state and Manaus reported 44,068 [3] and 9,058 [4] malaria cases, respectively.

The naturally acquired immunity against sexual forms of *P. vivax* in endemic regions remains unclear, as well as its interference with mosquitoes infectivity or “transmission-blocking effect” [5–7]. Transmission-blocking by antibodies against *Plasmodium* sexual forms has been increasingly becoming an important anti-malaria candidate strategy [8, 9]. Although gametocytemia has a key role in malaria transmission, specific factors associated with the presence, spatial and temporal patterns, and infectivity of these sexual forms to mosquitoes are not well understood. Molecular markers of gametocyte stages include *pfs25*/*pvs25*, which have been used successfully to detect gametocytes [10–13].

The potential transmission of the malaria parasite can be measured by *Anopheles* mosquitoes infection by gametocytes followed by detection of oocysts and sporozoites [14, 15]. Experimentally, mosquitoes can obtain a blood meal through a membrane [15, 16] or by biting infected individuals [17]. There are large discrepancies between the reported numbers of gametocytes and their infectivity. High densities do not necessarily result in infection whereas low densities can lead to infectivity [18].

Parasite infectivity is affected by vector competence and host’s immunity and genes [19]. The infectivity of *Plasmodium* gametocytes to *Anopheles* mosquitoes has been shown to be modulated by several molecules such as the antibody-like protein *pfs27*/*25* found in the sera of people exposed to malaria [20]. In addition to the relationship between mosquito infectivity and transmission-blocking immunity [21], complement system activation [22] and leukocytes [23] that phagocytose gametes inside the mosquito midgut shortly after the mosquito takes a blood meal [19, 24], a number of studies suggest a role for immune status in the transmission reduction of *P. falciparum* malaria [24, 25].

The presence of specific anti-*pfs48*/*45* and anti-*pfs230* monoclonal antibodies in the mosquito blood meal also results in transmission reduction [26]. The combination of all these specific hosts and vector factors, i.e. the vector immune system, the vector capacity, the relation of the mosquito infectivity and transmission block immunity, determine the variability and ability of gametocytemic individuals to infected mosquitoes.

Active detection of all parasite carriers and prompt treatment are critical to interrupt malaria transmission [27]. However, asymptomatic cases of malaria constitute a major challenge for elimination programs, especially regarding quantitation of parasitemia [28]. The use of a cut-off parasite density limit for the classification of asymptomatic infections and molecular tools have been suggested [29].

A cross-sectional study carried out in 2012 in the communities of Brasileirinho, Puraquequara, and Ipiranga and also analysed in the present study, showed that the prevalence of asymptomatic infection was 3.67% (74/2012), which corresponded to 85.06% (74/87) of the total *P. vivax* carriers (unpublished data). A 2002 study of native Amazonian populations showed that the prevalence of asymptomatic infections was 14.6, 21.7, and 6.4% in the three surveys at the same time, respectively, with a higher prevalence of asymptomatic infections in older age groups (odds ratio of 6.67 for people older than 40 years) [30]. Thus, the chances of presenting an asymptomatic infection increased significantly with age (Chi-square test for trend, *χ*^2^= 10.53, *P* = 0.001).

The main challenge of asymptomatic malaria is the identification of the individuals with low parasitemia which may constitute a parasite reservoir hindering malaria eradication efforts. Prevalence of asymptomatic cases of malaria varies considerably according to population age, previous exposure to malaria and probably also due to parasite and host immune factors [27, 31–33]. The natural protective immunity to *P. vivax* and *P. falciparum* infection in very low transmission is not understood in the context of current thinking about how natural immunity is acquired [34]. In the absence of evidence for the infectivity of these infections to mosquitoes, their role in and potential contribution to local malaria transmission remains unclear. This study thus aimed at understanding the role and impact of symptomatic and asymptomatic carriers on *P. vivax* transmission in the endemic region of Manaus in the western Brazilian Amazon.

## Methods

### Study area and patient recruitment

**Fig. 1:**
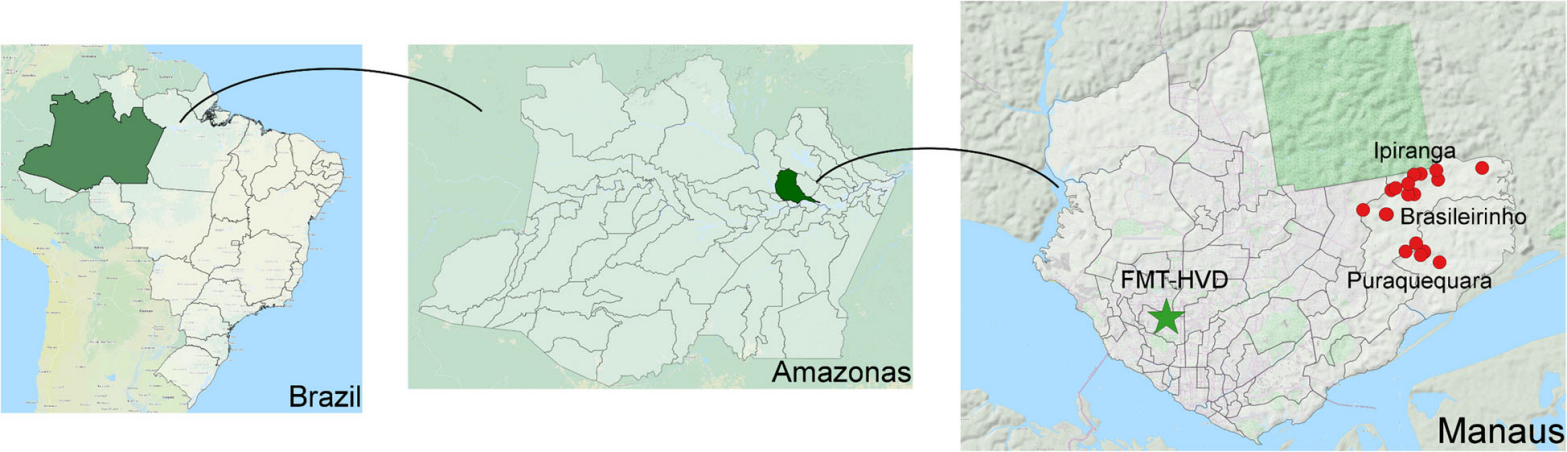
Hotspots of three communities of asymptomatic (red circles) and symptomatic (green star) malaria infections in the study area examined here. Samples from symptomatic and asymptomatic individuals were collected in the city of Manaus; symptomatic patients were all from Fundação de Medicina Tropical Dr Heitor Vieira Dourado (FMTHVD), a reference centre in tropical and infectious diseases, and the asymptomatic cases came from three peri-urban communities of the city

Here, we conducted a cross-sectional study. Symptomatic patients were recruited at the Fundação de Medicina Tropical Dr Heitor Vieira Dourado (FMT-HVD) between April 2013 and February 2015. Asymptomatic participants were recruited in the localities of Brasileirinho, Puraquequara, and Ipiranga, located in the periurban area of Manaus, state of Amazonas, Brazil (Fig. 1). Samples were collected in these areas from June 2014 to May 2015. Each locality has a full-time microscopy post, where the diagnosis of malaria is carried out. Daily, municipal health agents visit the residents through active and passive case detection. The clinical and parasitological characteristics of participants determined in this study are summarised in Table 1.

**Table 1:** Clinical aspects, infectivity mosquitoes and prevalence of asexual and sexual stages detected by microscopy and/or molecular biology tools in samples from symptomatic and asymptomatic malaria vivax patients

	Symptomatic (*n* = 42)	Asymptomatic (*n* = 25)
Male, *n* (%)	31 (74)	14 (56)
Age group (%)		
< 25	12	24
25–34	24	28
> 34	64	48
Mean	39	34.7
Malaria previous episodes (%)	88	84
Mean of malaria episodes	4	9
Symptoms (%)		
Fever	93	0
Headache	31	12
Chills	52	0
No. of dissected guts, mean ± SD (range)	27.6 ± 13.3 (11–85)	28.5 ± 13.1 (7–60)
Oocyst intensity, mean ± SD (range)	7.8 ± 18.1 (0–70)	2.1 ± 7.3 (0–35)
Parasite density by microscopy (parasites/μl), mean ± SD (range)		
Assexual forms, mean ± SD (range)	2454 ± 3148 (165–13,524)	249 ± 724 (0–3128)
Gametocytes, mean ± SD (range)	152 ± 130 (8–577)	13 ± 33 (0–151)
PCR parasite density (copies/μl)		
18S rDNA, mean ± SD (range)	14,134 ± 75,180 (1446–478,640)	2446 ± 5495 (0.7–20,627)
Pvs25, mean ± SD (range)	8937 ± 31,745 (505–175,571)	2711 ± 3444 (1–10,858)

*Abbreviation*: *SD* standard deviation

From symptomatic patients with positive thick blood smear, 2 × 5 ml blood samples were collected by venipuncture and stored at 37 °C until further processing. One heparinised sample was used to feed mosquitoes and the second (EDTA) tube was used for molecular procedures. Fifty μl of EDTA whole blood were transferred to RNAprotect (Qiagen, Hilden, Germany) within 1 h after sample collection. No patient had been on anti-malarial therapy during enrollment to the study. Data regarding age, sex, occupation, place of residence, and history of malaria were collected in a questionnaire. Fever (axillary temperature ≥ 37.5 °C at admission or history of fever in the last 48 h) and other signs and symptoms were assessed before blood collection. Tubes and questionnaires were individually bar-coded in the laboratory before blood collection.

Asymptomatic patients were diagnosed using 300 μl of whole blood collected with a fingerstick lancet and placed in a microtube containing anticoagulant (EDTA/sodium fluoride), the final concentration was 1.8mg/ ml. DNA was extracted from these samples using the FavorPrep^TM^ 96-well Genomic DNA Kit (Favorgen, Ping-Tung, Taiwan) and they were tested for the presence of *Plasmodium* spp. infections using a validated, genus-specific qPCR for the detection of *Plasmodium* spp. (Qmal assay) ( Additional file 1: Table S1, Additional file 2: Table S2) [35]. In case of a positive result for individuals who accepted to take part in the study, samples of venous blood were collected as described above. Individuals were considered asymptomatic when they did not present symptoms of malaria, thirty days before and thirty days after the blood collection to detect *P. vivax.*

### *Plasmodium vivax* peripheral parasitemia and gametocyte counts by light microscopy

Thick blood smears were Giemsa-stained and analysed by light microscopy for malaria parasite species and peripheral parasite counting by at least two independent trained microscopists. Inconsistencies were solved by a senior microscopist. The mean parasitemia was used. Parasite densities (parasites/μl) were calculated by counting the number of asexual and sexual parasites stages per 500 leukocytes.

### Assessment of *P. vivax* infectivity to mosquitoes

*Anopheles aquasalis* mosquitoes were reared under standard laboratory conditions at 27 °C with 80% humidity on a 12 h light/dark cycle. Mosquitoes were provided with 10% sucrose solution *ad libitum* until one day before the infective blood meal, as described previously [36, 37].

*Anopheles aquasalis* were infected with *P. vivax* by feeding blood collected from patients diagnosed with malaria using an artificial membrane feeding system [37]. Blood samples were maintained at 37 °C no later than one hour until membrane feeding assays. The second group was treated similarly but with inactivated-blood serum (IBS). The *P. vivax* infective blood samples were centrifuged for 1 min at 37 °C, and the serum was removed and heated for 1 h at 56 °C in a water bath to inactivate the complement system. In both groups, the mosquitoes were 3 to 5 days old (100 per cage for each group) and allowed to feed for 2 h in the dark. Afterwards, engorged females were kept with 10% sucrose daily until they were dissected on day 7 after feeding. The midguts were dissected in a drop of mercurochrome in phosphate-buffered saline, and oocysts were counted by examination under 10× light microscopy at 100× magnification (Additional file 3: Table S3).

### Molecular detection of asexual and sexual of *Plasmodium vivax*

EDTA blood was stored at 37 °C until samples processing. For RNA extraction, 50 μl of EDTA blood was transferred to 250 μl of RNAprotect transferred about 30 min after sample collection. The samples were immediately stored at -80 °C. A volume of 200 μl was stored for DNA isolation. The genomic DNA was extracted from the pelleted RBCs obtained from 200 μl blood using FavorPrep^TM^ 96-well Genomic DNA kit (Favorgen) according to the manufacturer’s instructions. RNA from whole blood stored in RNAprotect at -80 °C was extracted using the RNeasy® Plus 96 kit (Qiagen) as described [35].

Both the Qmal and species-specific qPCR assays target the 18S rRNA gene, as previously described [35, 38]. Data were analyzed using standard curves of dilutions of the plasmids containing the target sequences (kindly provided by I. Felger, Swiss TPH, Basel). C_q_ values (cycle threshold) of standard plasmids were used to determine the number of copies for the genes of interests for each sample. Analyses were performed by the software distributed by the manufacturer (Applied Biosystems' 7500 Fast System SDS Software, Waltham, Massachusetts, USA).

Plasmid dilutions were tested in quintuplicates. For the detection limit, three of the five dilutions had to be positive for *P. vivax:* 3 copies/μl, *pvs25*: 0.5 copy/μl [35]. The efficiency of the methodology tested in the laboratory LODs was for *P. vivax*: 1 copy/μl (efficiency 92.18%) and *pvs25*: 0.5 copy/μl (efficiency 96.20%) (Additional file 4: Table S4).

Detection of *P. vivax* gametocytes by RT-qPCR to detect *pvs25* transcripts of this gametocyte-specific gene was performed as previously described [35]. Plasmids served as positive controls and as standards for the quantification of DNA samples. Three dilutions used were: 10^2^, 10^4^ and 10^6^ copies/μl of the plasmids containing the gene fragment to be amplified. Analyses were performed using the Applied Biosystems software 7500 Fast System SDS Software.

### Statistical analysis

A person was defined as a transmitter if his/her blood sample had successfully infected at least one mosquito. A t-test was also used to compare the median percentage of mosquitoes infected by parasitemia group, and the Mann-Whitney *U-*test was used to compare the number of oocysts per infected midgut with parasitemia group. Relationships between the RT-qPCR quantification of gametocytes, gametocytemia, oocysts per infected midgut, and percentage of mosquitoes infected were compared using Pearson correlation analysis. Two-sided *P-*values < 0.05 were considered statistically significant. To assess whether gametocytemia would correctly classify mosquito infection, non-parametric receiver operating characteristic (ROC) analysis was performed. The area under the ROC curve (AUROC) was also calculated including its confidence intervals to measure the discriminative ability of gametocytemia as a predictor of mosquito infection.

## Results

### Study population

Sixty-seven participants were included in the study, of these 42 were symptomatic and 25 asymptomatic. The mean age of symptomatic patients was 39 years, and 88% of participants reported to have had at least one previous case of malaria (median: 3 episodes, range 1–20). The main symptoms reported by patients were fever (93%), chills (52%) and headache (31%). The mean parasitemia determined by light microscopy was 2454 parasites/μl (range 165–13,524) for asexual stages and 152 parasites/μl (range 8–577) for gametocytes, with the proportion of 83% (35/42). By qPCR, 40/42 patients were positive (95%) with an average 14,134 18S rRNA copies/μl (range 1446–478,640) and 8937 *pvs25* transcripts/μl (range 505–175,571) (Table 1).

At first 35 volunteers were enrolled as asymptomatic cases but ten of them were excluded as they showed symptoms within 15 days after recruitment, totalling 25 individuals for statistical analysis. The mean age of asymptomatic patients was 34.5 years with 85% of participants reporting having had at least one case of the disease (median: 9 episodes, range 1–43). The three (12%) asymptomatic individuals reported headaches. The mean parasite density as determined by light microscopy was 249 asexual stage parasites/μl (0–3128) and 13 gametocytes/μl (0–151). The average number of copies of 18S rRNA was 2446 (0.7–20,627), and 2711 (1–10,858) for the *pvs25* transcripts based on RT-qPCR analyses, in both cases 4 μl of blood volume were used (Table 1).

### Parasitemia and gametocytes detection

**Fig. 2:**
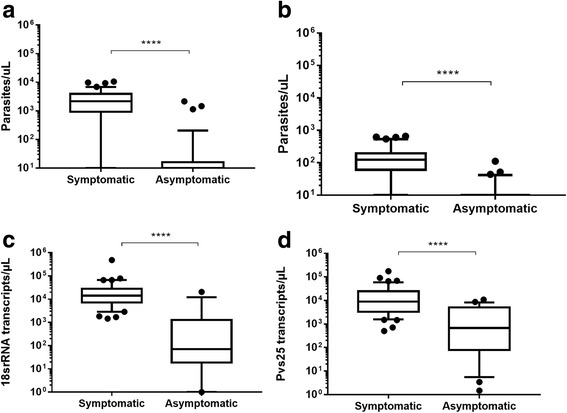
Parasitemia distribution in the symptomatic and asymptomatic groups. Semilogarithmic plots of asexual forms (**a**) and gametocytes (**b**) as determined by microscopy and parasite density estimated by the number of copies of 18S rRNA (**c**) and *pvs25* (**d**) are shown for symptomatic and asymptomatic blood samples. The symptomatic group showed the significantly higher density of both asexual forms **(a)** and gametocytes (**b**) (*P* < 0.0001) the parasite density was observed in the number of copies of 18S rRNA **(c)** and *pvs25* transcripts (**d)**. The means were compared using the Mann-Whitney test (****P < 0.0001), and the error bars represent the standard error of the mean (SEM)

By optical microscopy, 83% (35/42) of the symptomatic patients were positive for asexual and sexual stages of *P. vivax*. In asymptomatic patients only 40% (10/25) of analyzed samples were positive for *P. vivax* asexual stages and 24% (6/25) were positive for *P. vivax* gametocytes by light microscopy (Mann-Whitney U-test, *U*_(72)_ = 174, *Z* = 5.61, *P* < 0.0001 and *U*_(72)_ = 152, *Z* = 5.94, *P* < 0.0001, Fig. 2a and b, respectively). The symptomatic group showed significantly higher densitiy of number of copies of 18S rRNA and *pvs25* transcripts (Mann-Whitney U-test, *U*_(56)_ = 53, *Z* = 5.16, *P* < 0.0001 and *U*_(64)_ = 158, *Z* = 4.61, *P* < 0.0001, Fig. 2c and d).

Gametocytes detected by either microscopy and RT-qPCR showed 83% and 100% for symptomatic and 23% and 88% for asymptomatic infections, respectively.

**Fig. 3:**
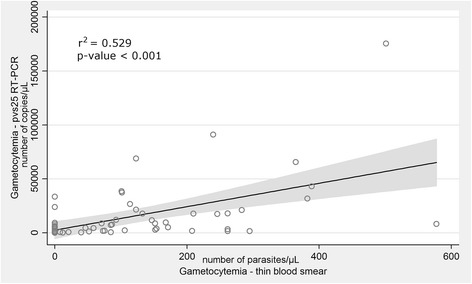
Correlation between gametocytes measured by levels of *pvs25 P. vivax* transcripts and gametocytes visualised by microscopy. A strong positive association between gametocyte numbers detected by RT-qPCR and those visualised by microscopy in thin blood smears from symptomatic individuals. The values indicate the correlation coefficient (*r*^2^) (*P*-value < 0.001)

A strong positive association for the gametocyte-detection techniques used (optical microscopy and *pvs25* transcripts measured by RTqPCR) was observed (*r*^2^_(61)_ = 0.529, *P* = 0.001, Fig. 3) highlighting a complementary useful approach in malaria diagnosis.

### Infectivity of asymptomatic patients

Asymptomatic patients had low parasitemias (Table 2), most of them submicroscopic, yet some were able to infect mosquitoes. Thirteen samples (50%) had negative slides for *P. vivax* asexual forms, 18 (69%) for gametocytes, and for two patients (8%), microscopy data were not available. In the asymptomatic group, the average infection rate was 1.42%, ranging from at least 0.91 to 24.24%; the average oocyst number for each sample ranged from 1–35 oocysts.

**Table 2:** Susceptibility of *An. aquasalis* mosquitoes to infection with *P. vivax* from Brazilian asymptomatic patients

Patient ID	Thin blood smear (parasites/μl)		PCR (copies/μl)		Infection rate (%)	Oocysts median (IQR)	Symptom-free days
	Asx	Gam	18 srRNA	Pvs25			
1	0	0	16	nd	24.24	25 (16–34)	ns
2	21	0	nd	6192	10	30 (-)	ns
3	0	0	nd	2072	12.5	35 (31.5–38.5)	ns
4	88	52	29	1263	0	0	7
5	0	0	51	1002	0	0	ns
6	nd	nd	62	595	0	0	ns
7	nd	nd	nd	10,858	5.71	1 (1–1)	ns
8	201	12	19	82	0	0	ns
9	3128	152	20,627	8686	14.89	12.25 (9–20)	10
10	2034	59	11,246	4393	5.12	7 (5.5–8.5)	1
11	77	0	97	240	0	0	ns
12	94	0	694	5190	0	0	14
13	0	0	186	252	0	0	30
14	0	0	1	23	0	0	4
15	0	0	1	147	1.69	1 (-)	1
16	301	40	58	262	0	0	ns
17	0	0	78	48	2.06	3.5 (2.75–4.25)	ns
18	0	0	nd	nd	0.91	1 (-)	ns
19	0	21	nd	757	0	0	5
20	211	0	nd	8	0	0	ns
21	0	0	nd	2	0	0	7
22	65	0	nd	4	0	0	ns
23	0	0	5005	4612	0	0	ns
24	0	0	82,849	7855	0	0	ns
25	0	0	2558	7817	0	0	ns

*Abbreviations*: *nd* not determined, *ns* no symptoms, *IQR* interquartile range (25th and 75th percentile), *SD* standard deviation, *(-)* single positive mosquito

**Fig. 4:**
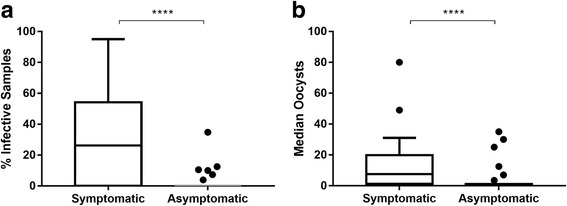
Susceptibility of *An. aquasalis* to infection with *P. vivax* from Brazilian symptomatic and asymptomatic patients. Infectivity to *An. aquasalis* mosquitoes (**a**) and oocysts (mean) at the midgut (**b**) are shown for symptomatic and asymptomatic blood samples used in MFAs. The means were compared using the Mann-Whitney test (****P < 0.0001), and the error bars represent the standard error of the mean (SEM)

Although the asymptomatic group with low submicroscopic parasitemia showed infectivity to mosquitoes, the two groups showed significant differences in infectivity and median oocysts by the (Mann-Whitney U-test, *U*_(72)_ = 280, *Z* = 4.66, *P* < 0.0001 and *U*_(66)_ = 267.5, *Z* = 3.99, *P* < 0.0001, Fig. 4a and b, respectively).

**Fig. 5:**
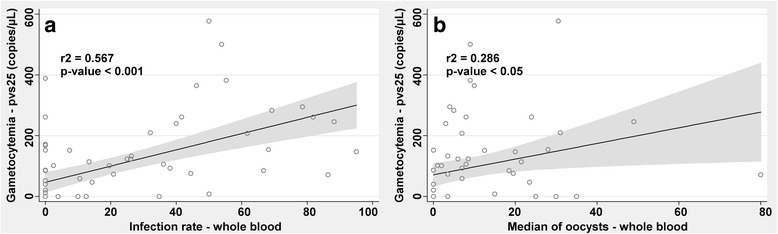
Correlation between gametocytes and infection rate and oocysts median. The number of mature gametocytes (measured by *pvs25* expression) is shown for MFAs performed with whole blood depending on the infection rate (**a**) and median of oocysts (**b**). The values indicate the correlation coefficient (*r*^2^) (*P* < 0.001 and *P* < 0.05)

A strong correlation was observed between *pvs25* transcripts and mosquito infection rate in the symptomatic and asymptomatic samples (*r*^2^_(69)_ = 0.567, *P* < 0.001, Fig. 5a) whereas the correlation was weaker between the median of oocysts and gametocytes in the same groups (*r*^2^_(61)_ = 0.286, *P* < 0.05, Fig. 5b).

**Fig. 6:**
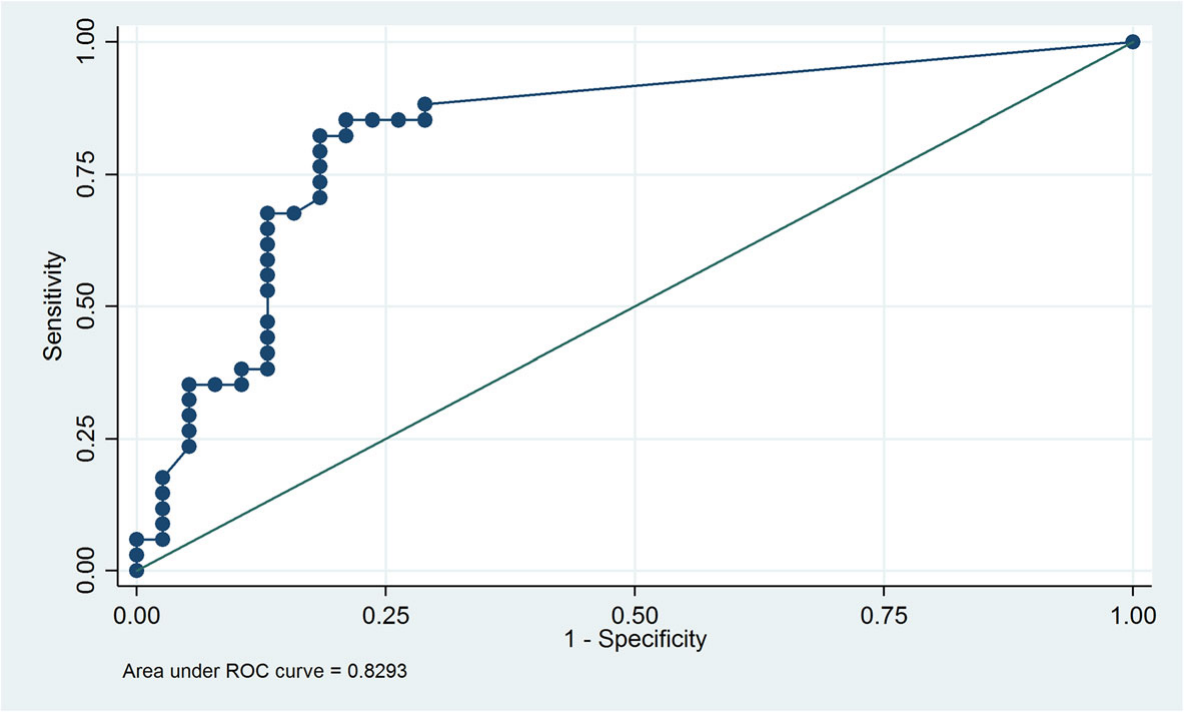
The ROC curve demonstrated through the sensitivity and specificity of *Anopheles aquasalis* to *P. vivax* infection. ROC curve of gametocytemia of the symptomatic and asymptomatic individuals as a predictor for mosquitoes infections; AUROC = 0.8293. The cut-off of 58.8 gametocytes/μl correctly classified 81.49% of mosquito infections

Furthermore, ROC analysis revealed that the geometric mean of gametocytes from symptomatic and asymptomatic individuals detected by light microscopy could be considered an acceptable classifier for mosquito infection (AUROC: 0.8293, CI: 0.73–0.93) (Fig. 6). A cut-off of 58.78 gametocytes/μl correctly classified 81.94% of the mosquito infections (sensitivity of 82.35%, specificity of 81.58%).

**Fig. 7:**
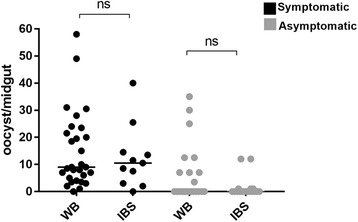
Susceptibility of *An. aquasalis* to infection with *P. vivax from* whole blood or inactivated blood serum. The number of oocysts per midgut of infected *An aquasalis* is shown in black for symptomatic and grey for asymptomatic individuals. The source of *P. vivax* parasites was either whole blood (WB) or inactivated blood serum (IBS). The medians were compared using the Mann-Whitney test (ns, not significant)

There was no significant difference in the production of oocysts when MFAs were set up with infective samples consisting of whole blood (WB) or inactivated blood serum (IBS) (Mann-Whitney U-test, *U*_(40)_ = 219*, Z* = 2.019, *P* = 0.9748, Fig. 7). Thus, inactivation of complement factors present in blood serum did not affect median oocysts as an indicator of infectivity.

## Discussion

Asymptomatic infections often go unnoticed and consequently untreated, resulting in important sources of gametocytes for local vectors. These infections and their contribution transmission are poorly understood, and there are few if any intervention that directly deals with them.

In this study, submicroscopic gametocyte carriage was common in an endemic malaria area in Manaus. Although these patients exhibited low parasitemia, their blood was nevertheless able to infect *Anopheline* mosquitoes. All parasitemia (asexual and sexual forms) rates were highest in symptomatic patients. Asymptomatics are showing a higher proportion of mature gametocytes (*pvs25* transcripts) were as effective as symptomatic in infecting mosquitoes (Fig. 5). A recent report from malaria-endemic regions in Colombia also showed the prevalence of *P. vivax* asymptomatic submicroscopic infections with mostly gametocytes as circulating parasites, of which 57% (8/14) were infective to *An. albimanus* mosquitoes when blood was used in MFAs [39].

A report from the Brazilian Amazon has shown that *P. vivax* can infect some mosquito species at higher rates with increasing number of gametocytes [*An. darlingi* (*Z* = -2.9, *P* < 0.01) and *An. aquasalis* (*Z* = -4.66, *P* < 0.001)] [37]. A report from Peru showed an increase in the number of gametocytes about the percentage of infected mosquitoes (*P* = 0.001); the number of oocysts also increased (*P* = 0.09) [19]. In a recent study with *An. dirus* in western Thailand a positive correlation was also found between the infection rate and gametocyte density (*P* = 0.003) or *pvs25* transcript abundance (*P* = 5 × 10^-6^). The mean oocyst density generally increased with blood parasite density (*P* = 2 × 10^-4^), gametocyte density (*P* = 4 × 10^-4^), and *pvs25* transcript abundance (*P* = 1 × 10^-8^) [31]. The odds of *P. vivax* transmission for samples with gametocytes were significantly higher than those with no gametocytes based on smear examination (OR: 6.35, 95% CI: 1.70–23.8, *P* = 0.003) [19].

The infection rate in this study was 41% for *An. aquasalis* when using blood from *P. vivax* symptomatic patients. In fact, the infection rate has been shown to be variable depending on the study area and *Plasmodium* species. In Manaus, Amazon, *An. albitarsis* (*s.l.*), *An. aquasalis*, *An. darlingi*, *An. nuneztovari* (*s.l.*) and *An. triannulatus* (*s.l.*), respectively, showed infection rates of 44.8%, 44.7%, 18.3%, 24.5% and 8.8% [37]. In western Thailand between 2014 and 2015, 84% (59/70) of symptomatic *P. vivax* blood samples were infective, i.e. at least one mosquito became infected with oocysts, and nearly 50% of all 4389 *An. dirus* mosquitoes became infected [31]. In Peru, the infection rate was 94% for *An. darlingi*/*P. vivax* [19]*.*

*Anopheles darlingi* and *An. aquasalis* are the most important malaria vectors in Brazil; previous studies have shown differences in infection rates for these species [35]. We observed a high production of oocysts by symptomatic patients compared to those produced by asymptomatic patients (Fig. 4b). *An. darlingi*, *An. albitarsis* (*s.l.*), *An. nuneztovari* (*s.l.*) and *An. triannulatus* (*s.l.*) field populations, and the laboratory-colonised *An. aquasalis* were shown to be susceptible to *P. vivax* under laboratory conditions with much higher infection rates than those reported in nature [37]. However, some studies have shown that mosquitoes infected in nature have few oocysts [40]. On average carriers of gametocytes at submicroscopic densities infected significantly fewer mosquitoes that in turn led to lower oocyst burdens [41–43].

Asymptomatic infections showing a higher gametocyte density (transcripts/μl) were as effective as symptomatic ones infecting mosquitoes. The ability to detect low levels of parasitemia is crucial to identifying asymptomatic carriers. Microscopy detected only 10 (40%), asymptomatic infections compared to the PCR-based method that was positive for all 25 samples. This increased sensitivity of molecular methods makes them suitable for identification of asymptomatic malaria [30, 42–44].

Symptomatic patients are promptly treated and remain infectious to mosquitoes only for a few days. Since asymptomatic patients not recognised become the main potentially infectious source for long periods, this can at least partially compensate for the low infectivity rate [43]. Thus, studying its role in transmission should be crucial for understanding infection [44].

Individuals who have had several previous episodes of symptomatic malaria are more likely to become asymptomatic carriers upon *Plasmodium* infection [45]. A higher number of previous cases of malaria were reported in asymptomatic patients in this study (Table 1), underscoring the importance of immunity in this population. Inactivation of the blood serum before mosquito feeding resulted in higher infection rates in *An. darlingi* and *An. triannulatus*, but not in *An. albitarsis* (*s.l.*) and *An. aquasalis* [37]. In the agreement, increased *An. aquasalis* mosquito infectivity was not observed in this study when using an inactive serum (Fig. 7). Therefore, the immune response underlying asymptomatic infection still needs to be elucidated.

## Conclusion

This study identified the potential of asymptomatic infection to contribute significantly to malaria transmission in the three communities studied in the city of Manaus. Individuals with submicroscopic parasitemias often do not present to health centres and become parasite reservoirs that go unnoticed by health surveillance and contribute to the endemicity of the disease in the Amazon region. Therefore, more studies are needed for this asymptomatic population to inform appropriate strategies as part of malaria control programs.

## Additional files

Additional file 1: **Table S1.** PCR setup of *Plasmodium*-specific qPCR (QMAL) and *P. vivax* specific qPCR based on detection of 18S rRNA genes and RT-qPCR detecting *pvs25* transcripts. For sequences of primers and probes see [35, 38]. (DOC 31 kb)

Additional file 2: **Table S2.** PCR cycling conditions used to detect parasites and gametocytes of *Plasmodium vivax* parasites. (DOC 32 kb)

Additional file 3: **Table S3.** Data from each membrane feeding assay of *Anopheles aquasalis* performed with samples from symptomatic and asymptomatic individuals of *Plasmodium vivax*. (DOC 151 kb)

Additional file 4: **Table S4.** Plasmids dilutions containing the sequence of the respective PCR product were used both as assay standards and to determine the limit of detection (LoD) of each assay. Generation of the plasmids is described in [35, 38]. (DOC 29 kb)

## Abbreviations

AUROC: Area under the ROC curve
C_q_: Cycle threshold
IBS: Inactivated blood serum
MFA: Membrane feeding assays
qPCR: Quantitative polymerase chain reaction
ROC: Receiver operating characteristic
RTqPCR: Reverse transcription quantitative polymerase chain reaction
WB: Whole blood
