# Supplementary material for: Infection of Anopheles aquasalis from symptomatic and asymptomatic Plasmodium vivax infections in Manaus, western Brazilian Amazon
Source: Parasit Vectors. 2018 May 4;11:288. doi: 10.1186/s13071-018-2749-0 (PMC5935932; doi:10.1186/s13071-018-2749-0)
Supplement: Supplementary file 1 — Table S1. PCR setup of Plasmodium-specific qPCR (QMAL) and P. vivax specific qPCR based on detection of 18S rRNA genes and RT-qPCR detecting pvs25 transcripts. For sequences of primers and probes see [35, 38]. (DOC 31 kb) [file 13071_2018_2749_MOESM1_ESM.doc]

Table S1: PCR setup of *Plasmodium*-specific qPCR (QMAL) and *P. vivax* specific qPCR based on detection of 18S rRNA genes and RT-qPCR detecting *pvs25* transcripts. For sequences of primers and probes see (Ref. #36 and #39).

| **Reagents** | **Concentration** | **Total Reaction Volume** | **DNA/RNA Volume** |
| --- | --- | --- | --- |
| QMAL, *P. vivax* qPCR |  |  |  |
| TaqMan® Gene Expression Master Mix (Applied Biosystems)  Primers Fw+Rev  Taqman® Probe | 2x  10µM  10µM | 12 µL | 4µL |
| *Pvs25* |  |  |  |
| TaqMan® 1-Step RT-PCR MasterMix (Applied Biosystems)  Primers Fw+Rev  Taqman Probe  TaqMan® Reverse Transcriptase Enzyme | 2x  10µM  10 µM  40x | 12 µL | 4µL |
