# Supplementary material for: Infection of Anopheles aquasalis from symptomatic and asymptomatic Plasmodium vivax infections in Manaus, western Brazilian Amazon
Source: Parasit Vectors. 2018 May 4;11:288. doi: 10.1186/s13071-018-2749-0 (PMC5935932; doi:10.1186/s13071-018-2749-0)
Supplement: Supplementary file 2 — Table S2. PCR cycling conditions used to detect parasites and gametocytes of Plasmodium vivax parasites. (DOC 32 kb) [file 13071_2018_2749_MOESM2_ESM.doc]

Table S2: PCR was carried out in an Applied Biosystems 7500 Fast Real-Time PCR System (Applied Biosystems) using the following conditions.

| **Step** | **Incubation** |
| --- | --- |
| **QMAL, *P. vivax* qPCR** |  |
| Pre-incubation | 50°C – 2 min |
| Initial Denaturation | 95°C – 10 min |
| Denaturation | 95°C- 15 sec |
| Annealing/Elongation | 58°C- 1 min |
| Number of Cycles | 45 |
| ***Pvs25*** |  |
| Reverse Transcription | 48°C – 15 min |
| Initial Denaturation | 95°C – 10 min |
| Denaturation | 95°C- 15 sec |
| Annealing/Elongation | 58°C- 1 min |
| Number of Cycles | 45 |
