# Supplementary material for: Infection of Anopheles aquasalis from symptomatic and asymptomatic Plasmodium vivax infections in Manaus, western Brazilian Amazon
Source: Parasit Vectors. 2018 May 4;11:288. doi: 10.1186/s13071-018-2749-0 (PMC5935932; doi:10.1186/s13071-018-2749-0)
Supplement: Supplementary file 3 — Table S3. Data from each membrane feeding assay of Anopheles aquasalis performed with samples from symptomatic and asymptomatic individuals of Plasmodium vivax. (DOC 151 kb) [file 13071_2018_2749_MOESM3_ESM.doc]

Table S3 – Data from each membrane feeding assay of *Anopheles aquasalis* performed with samples from Symptomatic and Asymptomatic individuals of *Plasmodium vivax*.

| **Patients** | **Symptomatic** | | | | | | | | | |
| --- | --- | --- | --- | --- | --- | --- | --- | --- | --- | --- |
|  | **Whole blood** | | | | | **Inactivated serum** | | | | |
|  | **Number of mosquitoes fed** | **Number of dissected gut** | **Number of infected**  **gut** | **Mean Oocyst Intensity** | **Mean Number of Oocyst (Min – Max)SD** | **Number of mosquitoes fed** | **Number of dissected gut** | **Number of infected gut** | **Mean Oocyst Intensity** | **Mean Number of Oocyst (Min – Max)SD** |
| **1** | 60 | 28 | 14 | 29 | (1 - 77) ±24 | ­ | ­ | ­ | ­ | ­ |
| **2** | 55 | 40 | 10 | 26.8 | (1 - 140) ±43 | ­ | ­ | ­ | ­ | ­ |
| **3** | 62 | 38 | 10 | 6.1 | (1 - 27) ±8 | ­ | ­ | ­ | ­ | ­ |
| **4** | 44 | 39 | 18 | 19.3 | (1 - 106) ±29 | ­ | ­ | ­ | ­ | ­ |
| **5** | 67 | 39 | 21 | 21.1 | (1 - 134) ±31 | ­ | ­ | ­ | ­ | ­ |
| **6** | 61 | 46 | 9 | 3.3 | (1 - 13) ±4 | ­ | ­ | ­ | ­ | ­ |
| **7** | 92 | 85 | 75 | 59.6 | (1 - 230) ±44 | ­ | ­ | ­ | ­ | ­ |
| **8** | 75 | 53 | 17 | 54 | (1 - 260) ±71 | ­ | ­ | ­ | ­ | ­ |
| **9** | 54 | 48 | 10 | 5.7 | (1 - 19) ±6 | ­ | ­ | ­ | ­ | ­ |
| **10** | 77 | 47 | 29 | 10.3 | (1 - 41) ±10 | ­ | ­ | ­ | ­ | ­ |
| **11** | 50 | 40 | 1 | 1 | 1 | ­ | ­ | ­ | ­ | ­ |
| **12** | 45 | 36 | 13 | 8.2 | (4 - 17) ±4 | ­ | ­ | ­ | ­ | ­ |
| **13** | 59 | 22 | 15 | 65 | (3 - 220) ±74 | ­ | ­ | ­ | ­ | ­ |
| **14** | 60 | 18 | 9 | 16.8 | (3 - 35) ±9 | ­ | ­ | ­ | ­ | ­ |
| **15** | 48 | 12 | 8 | 20.5 | (1 - 45) ±16 | ­ | ­ | ­ | ­ | ­ |
| **16** | 59 | 11 | 9 | 25.1 | (1 - 58) ±19 | ­ | ­ | ­ | ­ | ­ |
| **17** | 71 | 14 | 0 | 0 | 0 | ­ | ­ | ­ | ­ | ­ |
| **18** | 64 | 43 | 0 | 0 | 0 | 76 | 31 | 7 | 6.6 | (2 - 20) ±6 |
| **19** | 59 | 13 | 9 | 0 | 0 | 75 | 13 | 0 | 0 | 0 |
| **20** | 56 | 20 | 19 | 21.5 | (14 - 41) ±8 | 68 | 23 | 16 | 27 | (2 - 55) ±21 |
| **21** | 75 | 18 | 8 | 21.3 | (3 - 60) ±17 | 59 | 26 | 10 | 15.9 | (3 - 40) ±13 |
| **22** | 44 | 38 | 21 | 17.3 | (1 - 147) ±31 | 54 | 23 | 22 | 34.7 | (1 - 200) ±46 |
| **23** | 60 | 12 | 5 | 9.6 | (2 - 22) ±8 | 56 | 11 | 6 | 11.8 | (2 - 32) ±11 |
| **24** | 56 | 14 | 11 | 5 | (1 - 14) ±4 | - | ­ | ­ | ­ | - |
| **25** | 58 | 15 | 6 | 4.1 | (2 - 8) ±3 | - | ­ | ­ | ­ | - |
| **26** | 67 | 15 | 2 | 21.5 | (20 - 23) ±2 | 47 | 20 | 6 | 13.6 | (8 - 29) ±8 |
| **27** | 71 | 21 | 8 | 12 | (1 - 25) ±8 | 78 | 11 | 5 | 7.8 | (1 - 26) ±10 |
| **28** | 44 | 23 | 6 | 10 | (1 - 28) ±11 | 65 | 30 | 8 | 26.8 | (2 - 150) ±50 |
| **29** | 54 | 14 | 2 | 23.5 | (4 - 43) ±27 | 54 | 12 | 2 | 13.5 | (13 - 14) ±0.7 |
| **30** | 60 | 22 | 19 | 70 | (36 - 90) ±19 | 7857 | 20 | 5 | 44.4 | (28 - 60) ±13 |
| **31** | 48 | 20 | 0 | 0 | 0 | 80 | 18 | 0 | 0 | 0 |
| **32** | 69 | 22 | 0 | 0 | 0 | 46 | 26 | 0 | 0 | 0 |
| **33** | 78 | 30 | 0 | 0 | 0 | 87 | 32 | 0 | 0 | 0 |
| **34** | 55 | 25 | 0 | 0 | 0 | 57 | 31 | 0 | 0 | 0 |
| **35** | 46 | 18 | 0 | 0 | 0 | 50 | 16 | 0 | 0 | 0 |
| **36** | 62 | 44 | 0 | 0 | 0 | 65 | 45 | 0 | 0 | 0 |
| **37** | 58 | 35 | 0 | 0 | 0 | 77 | 30 | 0 | 0 | 0 |
| **38** | 69 | 30 | 0 | 0 | 0 | 56 | 18 | 0 | 0 | 0 |
| **39** | 76 | 42 | 0 | 0 | 0 | 47 | 20 | 0 | 0 | 0 |
| **40** | 57 | 35 | 0 | 0 | 0 | 67 | 24 | 0 | 0 | 0 |
| **41** | 43 | 22 | 0 | 0 | 0 | 43 | 36 | 0 | 0 | 0 |
| **42** | 78 | 28 | 0 | 0 | 0 | 71 | 41 | 0 | 0 | 0 |
| **Asymptomatic** | | | | | | | | | | |
| **1** | 60 | 23 | 8 | 24.7 | (12 - 36) ±9 | 57 | 10 | 0 | 0 | 0 |
| **2** | 58 | 10 | 1 | 30 | 30 | - | ­ | ­ | ­ | - |
| **3** | 55 | 16 | 2 | 35 | (28 - 42) ±10 | - | ­ | ­ | ­ | - |
| **4** | 58 | 18 | 0 | 0 | 0 | 45 | 19 | 0 | 0 | 0 |
| **5** | 79 | 28 | 0 | 0 | 0 | 67 | 24 | 0 | 0 | 0 |
| **6** | 85 | 30 | 0 | 0 | 0 | 78 | 30 | 0 | 0 | 0 |
| **7** | 79 | 15 | 0 | 0 | 0 | 88 | 20 | 2 | 1 | (1) |
| **8** | 66 | 21 | 0 | 0 | 0 | 86 | 24 | 0 | 0 | 0 |
| **9** | 47 | 27 | 2 | 12.5 | (6 - 19) ±9 | 57 | 20 | 5 | 16 | (9 - 30) ±9 |
| **10** | 89 | 19 | 2 | 7 | (4 - 10) ±4 | 54 | 20 | 0 | 0 | 0 |
| **11** | 67 | 28 | 0 | 0 | 0 | 62 | 33 | 0 | 0 | 0 |
| **12** | 71 | 21 | 0 | 0 | 0 | 73 | 34 | 0 | 0 | 0 |
| **13** | 59 | 18 | 0 | 0 | 0 | 65 | 8 | 0 | 0 | 0 |
| **14** | 63 | 7 | 0 | 0 | 0 | - | ­ | ­ | ­ | - |
| **15** | 78 | 17 | 0 | 0 | 0 | 80 | 42 | 1 | 1 | (1) |
| **16** | 68 | 35 | 0 | 0 | 0 | 78 | 58 | 0 | 0 | 0 |
| **17** | 73 | 51 | 2 | 3.5 | (2 - 5) ±2 | 65 | 46 | 0 | 0 | 0 |
| **18** | 85 | 60 | 0 | 0 | 0 | 94 | 49 | 1 | 1 | (1) |
| **19** | 70 | 45 | 0 | 0 | 0 | 76 | 40 | 0 | 0 | 0 |
| **20** | 86 | 51 | 0 | 0 | 0 | 69 | ­ | ­ | ­ | - |
| **21** | 77 | 40 | 0 | 0 | 0 | 75 | 40 | 0 | 0 | 0 |
| **22** | 73 | 31 | 0 | 0 | ± | 70 | 16 | 0 | 0 | ± |
| **23** | 57 | 27 | 0 | 0 | ± | 65 | 20 | 0 | 0 | ± |
| **24** | 68 | 23 | 0 | 0 | ± | 58 | 34 | 0 | 0 | ± |
| **25** | 76 | 37 | 0 | 0 | ± | 77 | 28 | 0 | 0 | ± |
