# Supplementary material for: Infection of Anopheles aquasalis from symptomatic and asymptomatic Plasmodium vivax infections in Manaus, western Brazilian Amazon
Source: Parasit Vectors. 2018 May 4;11:288. doi: 10.1186/s13071-018-2749-0 (PMC5935932; doi:10.1186/s13071-018-2749-0)
Supplement: Supplementary file 4 — Table S4. Plasmids dilutions containing the sequence of the respective PCR product were used both as assay standards and to determine the limit of detection (LoD) of each assay. Generation of the plasmids is described in [35, 38]. (DOC 29 kb) [file 13071_2018_2749_MOESM4_ESM.doc]

Table S4: Plasmids dilutions containing the sequence of the respective PCR product were used both as assay standards and to determine the limit of detection (LoD) of each assay. Generation of the plasmids is described in (Ref. #36 and #39).

| Assay | Efficiency | Slope | Y-Intercept | R² | LoD (plasmid copies/µl) |
| --- | --- | --- | --- | --- | --- |
| QMAL | 90.58 | -3.57 | 41.79 | 0.999 | 1 |
| *P. vivax* qPCR | 92.18 | -3.53 | 41.93 | 0.997 | 1 |
| *Pvs25* RT-qPCR | 96.20 | -3.42 | 36.09 | 0.999 | 0.5 |
